# Autoamputation of an ovarian mature cystic teratoma: a case report and a review of the literature

**DOI:** 10.1186/s12957-016-0981-7

**Published:** 2016-08-17

**Authors:** Keun Ho Lee, Min Jong Song, In Cheul Jung, Yong Seok Lee, Eun Kyung Park

**Affiliations:** Department of Obstetrics and Gynecology, College of Medicine, The Catholic University of Korea, 222 Banpo-daero Seocho-gu, Seoul, 06591 Republic of Korea

**Keywords:** Torsion, Ovarian teratoma, Autoamputation

## Abstract

**Background:**

Torsion is known to be the most frequent complication of ovarian teratomas. Torsion of the adnexa usually manifests with severe abdominal pain and is treated as an acute surgical emergency. However, it may be asymptomatic. Autoamputation of an ovary, along with other adnexal structures, due to previous torsion is extremely rare.

**Case presentation:**

A parasitic ovarian teratoma that underwent torsion, autoamputation, and reimplantation was found incidentally during laparoendoscopic single-site surgery (LESS). The amputated tumor was located in the omentum of the right upper abdomen of a patient with concomitant torsion of a left ovarian teratoma. The right ovary and tube were absent even though she had no surgical history. This finding could be interpreted as an autoamputation of the adnexa due to torsion of a previous ovarian cyst arising from the right ovary. We removed all masses by LESS.

**Conclusions:**

Although both ultrasonography and computed tomography were performed preoperatively in our patient, the correct diagnosis of autoamputation and exact localization of the teratoma were extremely difficult. Physicians should consider the possibility of an autoamputated ovarian cyst even if preoperative radiography shows no calcification.

## Background

Mature cystic teratomas (MCTs; dermoid cysts) occur most commonly in the ovary, with an incidence ranging from 5 to 25 % of all ovarian neoplasms [1]. Unilateral absence of the tube/ovary is an extremely rare condition occurring in only one of every 11,421 women [2]. The etiology of autoamputation is either congenital [3] or acquired, but is usually thought to be acquired, as these two structures have different embryologic origins [4]. In acquired cases of autoamputation, it mainly results from chronic torsion or a delay in the diagnosis of acute adnexal torsion. Torsion of the pedicle has been reported to be the most frequent complication of ovarian teratoma, occurring in 16.1 % of cases [1]. Ovarian autoamputation is a pathologic complication of ovarian torsion that may result in the formation of a parasitic ovarian teratoma. We will report on a patient who presented with coexistent torsion of a left ovarian teratoma and an ovarian teratoma in the omentum. The absence of the right adnexa and existence of ovarian tissue in the omental teratoma suggest that the tumor underwent autoamputation in this case. In February 2016, a PubMed search was performed using the search terms “ovarian teratoma,” “torsion,” “autoamputation,” “parasitic dermoid cyst,” “extragonadal omental teratoma,” and “ectopic ovary,” and a search of the collected literature was performed to identify previously reported cases. Cases were limited to the English literature and adult women. In conjunction with our case, we will present a literature review of autoamputation of ovarian teratomas, including 30 previously reported cases (Table 1).

**Table 1:** Autoamputation of ovarian mature cystic teratoma

Author, year Ref. number	Age	Size (cm)	Autoamputation localization	Side of ovary	Preoperative diagnosis	Ovarian tissue
Thornton K, 1881 [8]	NS	NS	Abdomen	Left ovary	Abdominal tumor	NS
Ekbladh LE, 1973 [22]	41	6 × 6	Omentum	Left ovary	NS	+
Huhn FO, 1975 [23]	28	Man’s hand	Broad ligament	NS	NS	+
Kearney MS, 1983 [24]	70	7 × 5 × 3.5	Omentum	Right ovary	Autopsy	+
Beyth Y, 1984 [25]	23	4 × 5	Abdomen	Right ovary	NS	NS
Compton AA, 1985 [26]	39	8 × 7	Omentum	Right ovary	Cholelithiasis	+
Ralls PW, 1987 [27]	45	5 × 5	Omentum	Left ovary	Pelvic mass	+
Leno C, 1987 [28]	66	8 × 6 × 6	Omentum	Left ovary	Pelvic mass	+
Smith R, 1990 [9]	68	6 × 5	Omentum	Right ovary	Pelvic mass	−
Kriplani A, 1995 [29]	36	7 × 6	Omentum	Both ovaries	Premature ovarian failure	+
Moon WJ, 1997 [30]	57	8 × 8 × 7	Omentum and liver	Right ovary	Abdominal mass	+
	53	16 × 8 × 13	Ascending colon and omentum	Right ovary	Abdominal mass	+
Ushakov FB, 1998 [7]	36	10 × 9	Mesenterium	Right ovary	Ovarian teratoma	+
	27	4 × 5	Near left ovary	Left ovary	Ovarian teratoma	+
Guleria K, 2002 [31]	50	10 × 10	Omentum and colon	Right ovary	Pelvic mass	+
Ollapallil J, 2002 [32]	46	10 × 4 × 3	Omentum	Left ovary	Abdominal mass	+
Pfitzman R, 2004 [33]	36	9 × 3	Omentum	Left ovary	Abdominal mass	+
Yoshida A, 2005 [34]	36	5 × 4 × 4	Omentum	Left ovary	Ovarian cyst	−
Kusaka M, 2007 [10]	24	5 × 4	Cul-de-sac	Left ovary	Ovarian cyst	+
Khoo CK, 2008 [35]	29	7 × 7	Pouch of Douglas	Right ovary	Ovarian cyst	NS
Moawad NS, 2008 [11]	59	4 × 2	Uterosacral ligament	Left ovary	Ovarian teratoma	+
Peitsidou A, 2009 [36]	33	8 × 5	Cul-de-sac	Right ovary	NS	+
Bartlett CE, 2009 [37]	29	8 × 6	Pouch of Douglas	Right ovary	Right adnexal mass	NS
Koga K, 2010 [38]	33	3 × 2	Peritoneal loose body	Left ovary	Pelvic mass	NS
Matsushita H, 2011 [39]	69	7	Cul-de-sac	Right ovary	Ovarian tumor	NS
Shetty NS, 2011 [40]	66	15 × 10	Right inguinal region	Right ovary	Right inguinal mass	+
Eda M, 2012 [41]	83	8.1	Pouch of Douglas	Left ovary	Pelvic mass	+
Koo YJ, 2012 [17]	34	4 × 3.2	Uterosacral ligament	Left ovary	Left ovarian teratoma	−
Kakuda M, 2015 [42]	41	4 × 3	Pouch of Douglas	Left ovary	Left ovarian teratoma	+
Chitrakar NS, 2015 [14]	33	11 × 11 × 6	Hepatorenal space	Right ovary	Abdominal teratoma	+
Current case, 2016	77	10 × 7	Omentum	Right ovary	Abdominal teratoma	+

*NS* not stated

## Case presentation

**Fig. 1:**
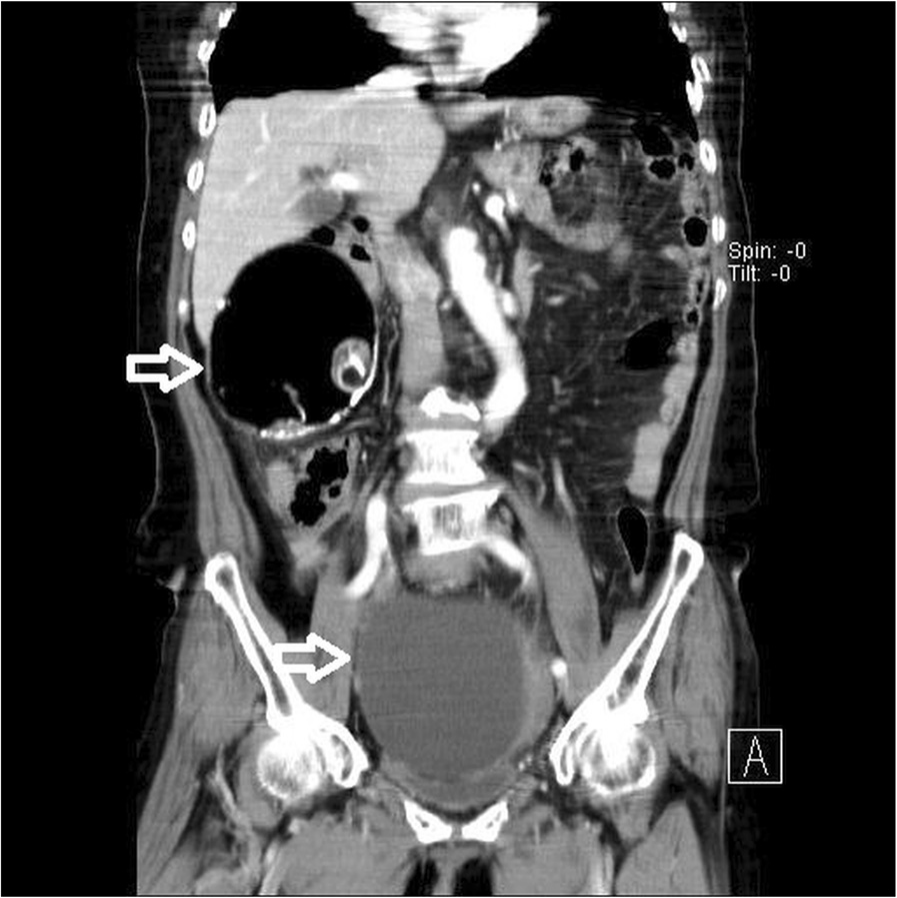
Abdominal computed tomography findings. The imaging indicates a mature cystic teratoma of the left ovary and right subhepatic space

**Fig. 2:**
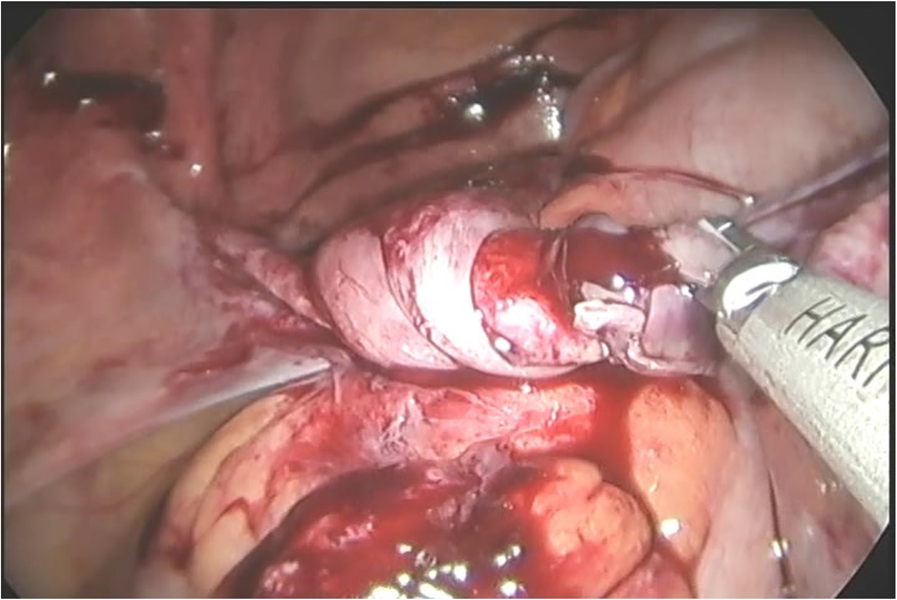
Laparoscopic findings. Torsion of the left adnexa with severe adhesion to the sigmoid colon is showed

**Fig. 3:**
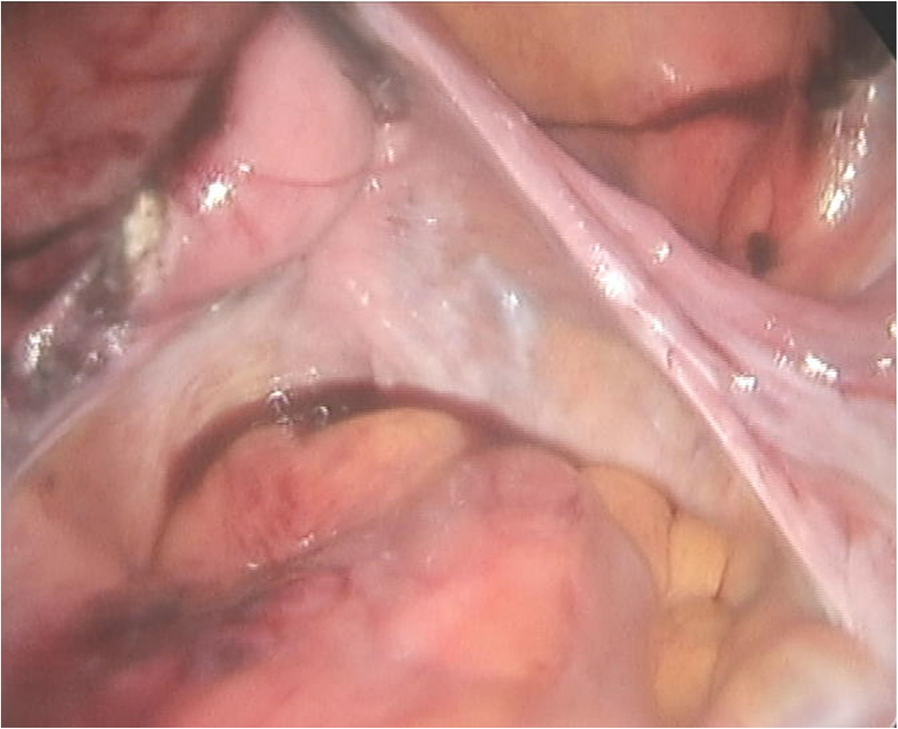
Laparoscopic findings. The right ovary and tube are absent

**Fig. 4:**
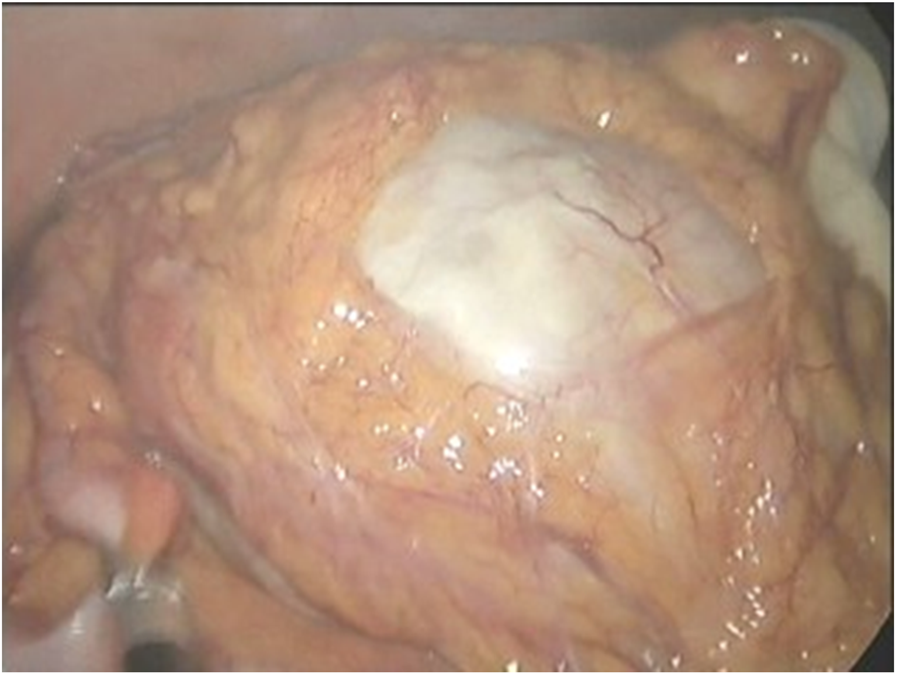
Laparoscopic findings. A second cystic mass found in the right subhepatic space is surrounded by the omentum, bowel, and appendix

A 77-year-old Korean woman, gravida 5, para 5, was admitted through the emergency room because of lower abdominal pain, poor oral intake, and a recent increase in abdominal size for 4 days. The symptoms had gradually increased in severity. She had no history of acute pain or previous operation. Abdominal examination revealed tenderness and rebound tenderness of the lower abdomen with a palpable mass in the left lower quadrant. Ultrasound examination showed an enlarged left ovarian cyst measuring 14.3 × 14 × 8.6 cm with diffuse internal echoes, including a 6.1 × 6.0 cm hypoechogenic component without significant vascularity. The right ovary was not seen and the uterus was normal and atrophied. Contrast-enhanced computed tomography (CT) revealed an approximately 12-cm well-circumscribed mass (of fat and soft tissue density) in the pelvic cavity and a 9-cm well-circumscribed mass (of fat and soft tissue density) with calcification in the right subhepatic space (Fig. 1). The suggested preoperative diagnosis was benign teratoma of the left ovary and right subhepatic space. With regard to preoperative examination, the laboratory tests, biochemical tests, complete blood counts, blood coagulation profile, and urinalysis were all normal. The C-reactive protein (CRP) level was elevated at 6.87 (normal range 0–0.3 mg/dL), CA-125 was elevated at 50.76 (normal range 0–35 U/mL), and CA 19-9 was normal at 22.06 (normal range 0–37 U/mL). We decided to proceed with laparoscopy. Laparoendoscopic single-site surgery (LESS) was performed through a 20-mm intraumbilical incision using a Glove port (NELIS, Bucheon, South Korea). During laparoscopy, torsion of the left adnexa due to an approximately 12 × 10 cm left ovarian cyst was visualized, with an atrophied normal uterus. The left adnexa was rotated 1440° clockwise with multifocal purple discoloration and severe adhesion to the sigmoid colon (Fig. 2). The right ovary and tube could not be identified in the proper anatomical location (Fig. 3). A second cystic mass of about 10 × 7 cm was noted in the right subhepatic space. It was surrounded by thin filmy adhesions to the omentum, bowel, and appendix (Fig. 4). Left adnexectomy, intra-abdominal mass excision, and appendectomy were performed by LESS. The abdominal mass was carefully dissected from the surrounding omentum and bowel using a monopolar hook dissector and harmonic scalpel (Ethicon, Somerville, NJ, USA). There was no pedicle that needed to be clamped and no identifiable blood supply. The mass was removed intact. The entire specimen was removed through the umbilical incision without leakage of content using an EndoBag (LapBag, Sejong Medical, Paju City, South Korea). The patient recovered uneventfully and was discharged 4 days after surgery. The histopathological examination confirmed MCTs in the left ovary and right subhepatic space. The abdominal mass included ovarian tissue. These findings could also be interpreted as an autoamputation of the adnexa due to torsion of a previous ovarian cyst arising from the right ovary.

## Conclusions

MCT is one of the most commonly found ovarian tumors, and 13.7 % of these tumors are bilateral [5]. Although teratomas most commonly occur in the ovaries, they have been documented in a number of other locations (0.4 % of all teratomas) [1]. Extragonadal teratomas occur anywhere along the midline of the body, such as the mediastinum, because of the migration of germ cells in embryonic life. However, parasitic or extragonadal teratomas in the abdominal cavity are extremely rare, and the most common extragonadal site is the omentum [6].

The causes of extragonadal teratomas are poorly understood, but several mechanisms have been proposed. It is generally known that teratomas arise from germ cells originating in mature gonads. Migration of germ cells from the yolk sac along the hindgut (mesenteric route) toward the genital ridge (primitive gonad) takes place during early fetal development. These totipotent cells (primordial germ cells or early embryonic cells) may give rise to a variety of tissues originating from the three primitive embryonic layers. Three proposed theories for extragonadal sites of teratoma exist: (1) autoamputation of an ovarian teratoma and reimplantation into an extragonadal site, (2) development in a supernumerary ovary, and (3) origination from displaced germ cells [7].

The first theory is that autoamputated extragonadal teratomas from an ovarian site may reimplant in another site in the abdominal cavity. This theory is widely accepted as the etiology of extragonadal teratomas in the abdominal cavity. Torsion of the pedicle has been reported to be the most frequent complication of ovarian teratomas. Autoamputation was first reported, along with possible mechanisms, by Thornton in 1881 [8]. The blood supply of the involved organ is impaired when torsion occurs, which may result in venous congestion and aseptic inflammation of the tumor wall. In an acute event, the tumor undergoes necrosis and subsequent atrophy due to ischemia. In a subacute or chronic event, the tumor may become adherent to adjacent structures with the formation of new collateral blood flow [7]. In a rare event, the tumor will become completely detached from the pedicle and become a parasitic teratoma. Although the mechanisms of autoamputation are still unclear, adnexal torsion is believed to lead to infarction, necrosis, and autoamputation. Theoretically, a parasitic ovarian teratoma results from torsion, autoamputation or detachment, and subsequent reimplantation or persists as a peritoneal loose body. The detachment of an ovarian teratoma may also occur without adnexal torsion. It has been suggested that adhesion formation with neovascularization between the tumor and the omentum may occur [9]. Table 1 contains a summary of the 30 reported cases of autoamputation of ovarian MCTs to date. In 15 cases, the teratomas were located in the omentum, and in 12 cases, they were located in the pelvis. The omentum, because of its special role in the intra-abdominal inflammation defense process and adhesion formation, is probably the primary location for reimplantation of the autoamputated ovary. The argument for autoamputation is especially compelling in the case of a unilaterally absent ovary [10, 11] and is supported by observation of an atrophic ovary or of ovarian stroma-like tissue in the tumor. However, it is not easy to distinguish autoamputation from other causes because the moment of autoamputation is not actually observed. In the reported cases, an ipsilateral absent or atrophic ovary was visualized in 29 patients. In histopathological evaluation of these cases, common features of parasitic teratoma included a cyst wall composed of bone tissue, containing adipose tissue and hair. Viable normal ovarian tissue could be seen with corpus albicans and thin ovarian stroma. In 21 cases, the parasitic teratoma included microscopic ovarian tissue, in six cases, it was not stated, and in two cases, the teratoma did not contain ovarian tissue (Table 1). The alternative fate of the amputated adnexa of phagocytosis, organization, and eventual reabsorption makes it difficult to discern the state of the affected adnexa prior to amputation. In the present case, the parasitic teratoma contained ovarian tissue, and the absence of the right adnexa was observed on laparoscopy. In cases of congenital absence of the adnexa, renal anomalies are often observed because müllerian structures and gonads arise from different embryologic origins. However, our patient did not have renal anomalies. Thus, autoamputation may be a reasonable theory in our case, although it is difficult to prove.

The second theory is that extragonadal teratomas may occur in an ectopic ovary [12], which is thought to arise congenitally or following surgery or pelvic inflammation. Our patient had no history of laparotomy, and the tumor contained ovarian tissues. Therefore, this theory may be applicable if the right adnexa was absent congenitally.

The third theory posits that extragonadal teratomas may originate from displaced primordial germ cells [13]. Primordial germ cells may stop differentiating during migration, thus later causing a teratoma. However, this theory does not appear to be applicable because the teratoma contained viable ovarian tissue in our case.

Following reports by Moawad [11] and Chitrakar [14], our case is now the third report of a parasitic ovarian teratoma in the omentum with the absence of the right adnexa and concurrent torsion of a left ovarian teratoma. Previous reports were confirmed by conventional laparoscopy or laparotomy.

The ovary and fallopian tube are extremely mobile structures that are normally able to tolerate up to 90° of rotation; rotation beyond this point increases the risk of vascular impairment. There is a tendency for abnormal rotation with a long fallopian tube or absent mesosalpinx. The size and weight of large ovarian cysts may cause elongation of the broad ligament; thus, the broad ligament acts as a fulcrum about which the ovary can twist. It has been postulated that torsion may occur more frequently with the right ovary due to hypermobility of the cecum and ileum (approximately 60 % of cases of torsion), presumably because of the proximity of the left ovary to the sigmoid colon, which can serve as a barrier [7]. Upon review, the right adnexa, as in our case, was involved in 15 of 29 cases and the left adnexa in 14 of 29 cases, as shown in Table 1. Tumor sizes varied, ranging from 5 to 16 cm for the right ovary and from 3 to 9 cm for the left ovary. Torsion of the adnexa seems to be related to multifocal factors such as the length of the tube, size of the ovarian cyst, adhesion of the sigmoid colon, and inflammation. Ovarian tumors, both benign and malignant, are implicated in 50–60 % of cases of torsion. The involved masses were nearly all larger than 4–6 cm, although torsion is still possible with smaller masses [4]. Malignant tumors are much less likely to result in torsion than benign tumors. This is because of the presence of cancerous adhesions that fix the ovary to surrounding tissues. Postmenopausal women with an adnexal mass or adolescents may be affected by torsion (about 17 % of cases); this may be because of changes in the weight of the adnexae [15]. Postmenopausal women with ovarian teratomas need surgical intervention to avoid torsion. In patients who need to retain vital reproductive function, we recommend prophylactic diagnostic laparoscopy in patients with significant symptoms or a large cystic lesion (>4 cm) to decrease the risks of torsion and subsequent autoamputation.

In a review of the literature for adnexal torsion [16], researchers recommended a laparoscopic approach over laparotomy and detorsion in all premenopausal women. They also recommended removing any associated adnexal masses during detorsion to prevent recurrence. Owing to the lack of long-term data, they did not recommend any fixation procedures like oophoropexy.

In seven of 30 cases, the teratoma was removed by conventional laparoscopic surgery, and in one case, a teratoma of the uterosacral ligament was treated with LESS [17]. Since 2011, our hospital has performed LESS for the evaluation of benign or early malignant ovarian masses. Therefore, we performed LESS for teratoma removal in our patient. There is considerable literature to suggest that LESS has potential advantages over conventional laparoscopic surgery. In a recent study, LESS was both feasible and comparable in surgical outcomes to conventional laparoscopic surgery for women with an ovarian MCT [18]. LESS was associated with less postoperative pain and required less analgesia. In our experience, LESS is a more suitable technique for ovarian teratoma than other ovarian cysts. The contents of ovarian teratoma include hair follicles, cartilage, fat tissue, and bony tissue. Extraction of that tissue through the 10-mm port used during conventional laparoscopy has some limitations and LESS has some advantages in this aspect, although further study is needed.

Malignant transformation (MT) is very rare, occurring in only 0.17–2 % of mature teratoma cases. The most frequent malignancy arising in mature teratomas is squamous cell carcinoma (SCC) (88.3 %), followed by adenocarcinoma, fibrosarcoma, carcinoid tumor, and mixed tumors [19]. MT usually occurs in postmenopausal women and may be associated with high tumor diameter [20]. In postmenopausal women, it is possible that prolonged exposure to various carcinogens in the pelvic cavity might lead to malignant transformation. Tumors with a diameter >10 cm are associated with high risk of malignancy in some studies and the MT of MCTs was quite high when the patient was >40 years with a serum SCC antigen over 2.5 ng/mL [21]. Our patient was at high risk of MT as a postmenopausal woman with a large tumor, although it is rare. SCC test and intraoperative frozen section could not be performed in the emergency setting. Preoperative imaging studies indicated benign MCTs and we planned for adnexectomy, considering the age of the patient and avoiding spillage of cystic content. We believe that the application of LESS is acceptable for this type of tumor in general.

As ultrasonography is now widely available as a valuable tool for the diagnosis of ovarian tumors, it may also assist preoperative diagnosis of ovarian torsion in some cases [12]. However, chronic adnexal torsion with no clinical signs and symptoms is difficult to diagnose preoperatively. In 31 cases, including our own, preoperative diagnosis of the autoamputated ovary was not achieved; all cases were diagnosed during an exploratory laparotomy (22 cases) or laparoscopy (nine cases), including our case. Physicians should recall the possibility of an autoamputated ovarian cyst even if preoperative ultrasonography and computed tomography show no calcification, and should perform prompt diagnostic laparoscopy.

In conclusion, because the parasitic teratoma specimen included ovarian tissue and the right adnexa was absent in our case, we concluded that the teratoma resulted from the autoamputation of a right ovarian teratoma. Our patient had no history of acute pain that was suspicious for previous adnexal torsion. Ovarian autoamputation is a rare clinical entity with uncertain etiology and may be asymptomatic on a number of occasions. Preoperative diagnosis of autoamputation is difficult. If intraperitoneal teratoma is suspected on an imaging study, physicians should consider the possibility of adnexal autoamputation, including asymptomatic torsion. It is important that all physicians treating female patients of reproductive age, especially with a pelvic mass and lower abdominal pain, be aware of the possibility of adnexal torsion with regard to fertility and ovary preservation. If symptoms persist, early diagnostic laparoscopy for exact diagnosis and therapy is recommended. In these cases, LESS may have an advantage for teratoma extraction.

## Abbreviations

CT: Computed tomography
LESS: Laparoendoscopic single-site surgery
MCTs: Mature cystic teratomas
MT: Malignant transformation
SCC: Squamous cell carcinoma

## References

[1] Peterson WF, Prevost EC, Edmunds FT, Hundley JM, Morris FK (1955). Benign cystic teratomas of the ovary; a clinico-statistical study of 1,007 cases with a review of the literature. Am J Obstet Gynecol.

[2] Sivanesaratnam V (1986). Unexplained unilateral absence of ovary and fallopian tube. Eur J Obstet Gynecol Reprod Biol.

[3] Eustace DL (1992). Congenital absence of fallopian tube and ovary. Eur J Obstet Gynecol Reprod Biol.

[4] Focseneanu MA, Omurtag K, Ratts VS, Merritt DF (2013). The auto-amputated adnexa: a review of findings in a pediatric population. J Pediatr Adolesc Gynecol.

[5] Ayhan A, Aksu T, Develioglu O, Tuncer ZS, Ayhan A (1991). Complications and bilaterality of mature ovarian teratomas (clinicopathological evaluation of 286 cases). Aust N Z J Obstet Gynaecol.

[6] Hegde P (2014). Extragonadal omental teratoma: a case report. J Obstet Gynaecol Res.

[7] Ushakov FB, Meirow D, Prus D, Libson E, BenShushan A, Rojansky N (1998). Parasitic ovarian dermoid tumor of the omentum—a review of the literature and report of two new cases. Eur J Obstet Gynecol Reprod Biol.

[8] Thornton KD (1881). Dermoid cyst. Am J Obstet.

[9] Smith R, Deppe G, Selvaggi S, Lall C (1990). Benign teratoma of the omentum and ovary coexistent with an ovarian neoplasm. Gynecol Oncol.

[10] Kusaka M, Mikuni M (2007). Ectopic ovary: a case of autoamputated ovary with mature cystic teratoma into the cul-de-sac. J Obstet Gynaecol Res.

[11] Moawad NS, Starks D, Ashby K (2008). Ectopic ovarian teratoma of the uterosacral ligament associated with a large ovarian dermoid. J Minim Invasive Gynecol.

[12] Sinha R, Sundaram M, Lakhotia S (2009). Multiple intraabdominal parasitic cystic teratomas. J Minim Invasive Gynecol.

[13] Oosterhuis JW, Stoop H, Honecker F, Looijenga LH (2007). Why human extragonadal germ cell tumours occur in the midline of the body: old concepts, new perspectives. Int J Androl.

[14] Chitrakar NS, Suwal S, Neupane S (2015). Bilateral ovarian teratoma: one parasitic twisted in-situ and another parasitic at the hepato renal space. J Nepal Health Res Counc.

[15] Melcer Y, Sarig-Meth T, Maymon R, Pansky M, Vaknin Z, Smorgick N (2016). Similar but different: a comparison of adnexal torsion in pediatric, adolescent, and pregnant and reproductive-age women. J Womens Health (Larchmt).

[16] Sasaki KJ, Miller CE (2014). Adnexal torsion: review of the literature. J Minim Invasive Gynecol.

[17] Koo YJ, Im KS, Jung HJ, Kwon YS (2012). Mature cystic teratoma of the uterosacral ligament successfully treated with laparoendoscopic single-site surgery. Taiwan J Obstet Gynecol.

[18] Park JY, Kim DY, Suh DS, Kim JH, Nam JH (2015). Laparoendoscopic single-site versus conventional laparoscopic surgery for ovarian mature cystic teratoma. Obstet Gynecol Sci.

[19] Brudie LA, Khan F, Radi MJ, Yates MM, Ahmad S (2016). Malignant melanoma arising in a mature teratoma: a case report with review of the recent literature. Gynecol Oncol Rep.

[20] Koc S, Tapisiz OL, Turan T, Ocalan R, Ozfuttu A, Boran N (2015). Malignant transformation of mature cystic teratoma of the ovary: a case series. J Exp Ther Oncol.

[21] Hackethal A, Brueggmann D, Bohlmann MK, Franke FE, Tinneberg HR, Munstedt K (2008). Squamous-cell carcinoma in mature cystic teratoma of the ovary: systematic review and analysis of published data. Lancet Oncol.

[22] Ekbladh LE, Fishburne JI (1973). Parasitized dermoid cyst of the omentum. Obstet Gynecol.

[23] Huhn FO (1975). Dermoid cysts of the greater omentum (author’s transl). Arch Gynakol.

[24] Kearney MS (1983). Synchronous benign teratomas of the greater omentum and ovary. Case report. Br J Obstet Gynaecol.

[25] Beyth Y, Bar-On E (1984). Tuboovarian autoamputation and infertility. Fertil Steril.

[26] Compton AA, Tandan A, Fleming WP (1985). Coexistent benign teratomas of the omentum and ovary. A case report. J Reprod Med.

[27] Ralls PW, Hartman B, White W, Radin DR, Halls J (1987). Computed tomography of benign cystic teratoma of the omentum. J Comput Assisted Tomogr.

[28] Leno C, Combarros O, Berciano J (1987). Lumbosacral plexopathy due to dermoid cyst of the greater omentum. Postgrad Med J.

[29] Kriplani A, Takkar D, Karak AK, Ammini AC (1995). Unexplained absence of both fallopian tubes with ovary in the omentum. Arch Gynecol Obstet.

[30] Moon W, Kim Y, Rhim H, Koh B, Cho O (1997). Coexistent cystic teratoma of the omentum and ovary: report of two cases. Abdom Imaging.

[31] Guleria K, Sahu B, Suneja A, Yadav P, Agarwal N (2002). Parasitic ovarian dermoid tumour. Aust N Z J Obstet Gynaecol.

[32] Ollapallil J, Werapitiya SB, Irukulla S, Gunawardena ID (2002). Benign cystic teratoma of the omentum. ANZ J Surg.

[33] Pfitzmann R, Klupp J, Krenn V, Neuhaus P (2004). A dermoid cyst in the greater omentum as a rare epigastric tumor. Z Gastroenterol.

[34] Yoshida A, Murabayashi N, Shiozaki T, Okugawa T, Tabata T (2005). Case of mature cystic teratoma of the greater omentum misdiagnosed as ovarian cyst. J Obstet Gynaecol Res.

[35] Khoo CK, Chua I, Siow A, Chern B (2008). Parasitic dermoid cyst of the pouch of Douglas: a case report. J Minim Invasive Gynecol.

[36] Peitsidou A, Peitsidis P, Goumalatsos N, Papaspyrou R, Mitropoulou G, Georgoulias N (2009). Diagnosis of an autoamputated ovary with dermoid cyst during a Cesarean section. Fertil Steril.

[37] Bartlett CE, Khan A, Pisal N (2009). Parasitic dermoid cyst managed laparoscopically in a 29-year-old woman: a case report. J Med Case Rep.

[38] Koga K, Hiori H, Osuga Y, Nagai M, Yano T, Taketani Y (2010). Autoamputated adnexa presents as a peritoneal loose body. Fertil Steril.

[39] Matsushita H, Kurabayashi T, Yanase T, Hashidate H (2009). Autoamputation of an ovarian cyst: a case report. J Reprod Med.

[40] Shetty NS, Vallabhaneni S, Patil A, Babu MM, Baig A (2013). Unreported location and presentation for a parasitic ovarian dermoid cyst in an indirect inguinal hernia. Hernia.

[41] Eda M, Kaidoh T, Takanashi Y, Inoue T (2012). A stone-like ovarian dermoid cyst in the Douglas’ Pouch of an elderly woman. Pathol Int.

[42] Kakuda M, Matsuzaki S, Kobayashi E, Yoshino K, Morii E, Kimura T (2015). A case of extragonadal teratoma in the pouch of Douglas and literature review. J Minim Invasive Gynecol.

